# The genome of *Rhizobium leguminosarum *has recognizable core and accessory components

**DOI:** 10.1186/gb-2006-7-4-r34

**Published:** 2006-04-26

**Authors:** J Peter W Young, Lisa C Crossman, Andrew WB Johnston, Nicholas R Thomson, Zara F Ghazoui, Katherine H Hull, Margaret Wexler, Andrew RJ Curson, Jonathan D Todd, Philip S Poole, Tim H Mauchline, Alison K East, Michael A Quail, Carol Churcher, Claire Arrowsmith, Inna Cherevach, Tracey Chillingworth, Kay Clarke, Ann Cronin, Paul Davis, Audrey Fraser, Zahra Hance, Heidi Hauser, Kay Jagels, Sharon Moule, Karen Mungall, Halina Norbertczak, Ester Rabbinowitsch, Mandy Sanders, Mark Simmonds, Sally Whitehead, Julian Parkhill

**Affiliations:** 1 Department of Biology, University of York, York, UK; 2 The Wellcome Trust Sanger Institute, Wellcome Trust Genome Campus, Cambridge, UK; 3 School of Biological Sciences, University of East Anglia, Norwich, UK; 4 School of Biological Sciences, University of Reading, Reading, UK

## Abstract

**Background:**

*Rhizobium leguminosarum *is an α-proteobacterial N_2_-fixing symbiont of legumes that has been the subject of more than a thousand publications. Genes for the symbiotic interaction with plants are well studied, but the adaptations that allow survival and growth in the soil environment are poorly understood. We have sequenced the genome of *R. leguminosarum *biovar *viciae *strain 3841.

**Results:**

The 7.75 Mb genome comprises a circular chromosome and six circular plasmids, with 61% G+C overall. All three rRNA operons and 52 tRNA genes are on the chromosome; essential protein-encoding genes are largely chromosomal, but most functional classes occur on plasmids as well. Of the 7,263 protein-encoding genes, 2,056 had orthologs in each of three related genomes (*Agrobacterium tumefaciens*, *Sinorhizobium meliloti*, and *Mesorhizobium loti*), and these genes were over-represented in the chromosome and had above average G+C. Most supported the rRNA-based phylogeny, confirming *A. tumefaciens *to be the closest among these relatives, but 347 genes were incompatible with this phylogeny; these were scattered throughout the genome but were over-represented on the plasmids. An unexpectedly large number of genes were shared by all three rhizobia but were missing from *A. tumefaciens*.

**Conclusion:**

Overall, the genome can be considered to have two main components: a 'core', which is higher in G+C, is mostly chromosomal, is shared with related organisms, and has a consistent phylogeny; and an 'accessory' component, which is sporadic in distribution, lower in G+C, and located on the plasmids and chromosomal islands. The accessory genome has a different nucleotide composition from the core despite a long history of coexistence.

## Abstract 2

The genome sequence of the α-proteobacterial N2-fixing symbiont of legumes, *Rhizobium leguminosarum*, is described, revealing a 'core' and an 'accessory' component.

## Background

The symbiosis between legumes and N_2_-fixing bacteria (rhizobia) is of huge agronomic benefit, allowing many crops to be grown without N fertilizer. It is a sophisticated example of coupled development between bacteria and higher plants, culminating in the organogenesis of root nodules [1]. There have been many genetic analyses of rhizobia, notably of *Sinorhizobium meliloti *(the symbiont of alfalfa), *Bradyrhizobium japonicum *(soybean), and *Rhizobium leguminosarum*, which has biovars that nodulate peas and broad beans (biovar *viciae*), clovers (biovar *trifolii*), or kidney beans (biovar *phaseoli*).

The Rhizobiales, an α-proteobacterial order that also includes mammalian pathogens *Bartonella *and *Brucella *and phytopathogenic *Agrobacterium*, have diverse genomic architectures. The single chromosome of *Bartonella *is small (1.6-1.9 Mb [2]), but the larger (approximately 3.3 Mb) *Brucella *genomes comprise two circles [3-5]. Genomes of the plant-associated bacteria are larger still; that of *A. tumefaciens *is about 5.6 Mb, with one circular and one linear chromosome, plus two native plasmids [6,7]. To date, three rhizobial genomes have been sequenced. *S. meliloti *1021 has a 3.5 Mb chromosome plus two megaplasmids, namely pSymA (1.35 Mb) and pSymB (1.68 Mb), with the former having genes for nodulation (*nod*) and symbiotic N_2 _fixation (*nif *and *fix*) [8]. In contrast, the symbiosis genes of *Mesorhizobium loti *MAFF303099 (which nodulates *Lotus*) and of *B. japonicum *USDA110 are on chromosomal 'symbiosis islands', with the chromosome of the latter (9.1 Mb) being among the largest yet known in bacteria [9,10].

*Rhizobium leguminosarum *has yet another genomic architecture: one circular chromosome and several large plasmids, the plasmid portfolio varying markedly among isolates in terms of sizes, numbers, and incompatibility groups [11-14]. The subject of the present study, *R. leguminosarum *biovar *viciae *(Rlv) strain 3841 (a spontaneous streptomycin-resistant mutant of field isolate 300 [15,16]), has six large plasmids; pRL10 is the pSym (symbiosis plasmid) and pRL7 and pRL8 are transferable by conjugation [17].

The distinction between 'chromosome' and 'plasmid' has become blurred in recent years with the discovery that many bacteria have more than one replicon with over a million base pairs. For example, the second replicon of *Brucella melitensis *16M is called a chromosome (1.18 Mb) [3], whereas the equivalent in *S. meliloti *1021 is referred to as a megaplasmid (pSymB; 1.68 Mb) [8]. They both replicate using the *repABC *system as is typical of plasmids, and both carry the only copies of certain essential genes, although the *B. melitensis *chromosome II has many more of these as well as a complete ribosomal RNA operon. What combination of size, replication system, rRNA genes, and essentiality should qualify a replicon to be called a chromosome is probably more a matter of semantics than of biology.

A more important distinction, in our view, is between 'core' and 'accessory' genomes. This distinction predates the genomics era; indeed, it has been discussed for more than a quarter of a century. Davey and Reanney [18] contrasted 'universal' and 'peripheral' genes, or 'conserved' and 'experimental' DNA. Campbell [19] wrote of 'euchromosomal' and 'accessory' DNA and explained how gene transfer was important in shaping the latter. He pointed out that genes carried by plasmids or transposons were 'available to all cells of the species, though not actually present in them' and 'should typically be genes that are needed occasionally rather than continually under natural conditions'. Furthermore, the need to function in different genetic backgrounds meant that 'evolution must limit the development of specific interactions between their products and those of universal genes'. This would tend to sharpen the separation between the euchromosomal and accessory gene pools, although transfer between them would remain possible.

The expectation is that particular accessory genes will often be absent from closely related strains or species, and as comparative data became available such genes were indeed found in large numbers [20]. They often had a nucleotide composition different from the bulk of the genome, and this property had previously been interpreted as evidence that they were 'foreign' genes [21]. This is plausible because nucleotide composition can be quite different between distantly related bacteria even though it is relatively consistent within genomes [22]. This pattern is thought to reflect biased mutation rates that tend to create a distinctive composition for each genome [23], and if these 'foreign' genes remained long enough they would gradually ameliorate toward the local composition [20]. Unusual composition is not an infallible indicator of recent acquisition [24], although there is a strong tendency for genes acquired by *Escherichia coli *to be A+T rich [25]. Amelioration will be expected if genes of unusual composition are normal genes that reflect the composition of some distant donor species [20], but an alternative explanation is that they represent a class of genes that maintain a distinct composition. Daubin and coworkers [26] pointed out that phage and insertion sequences are generally A+T rich, and suggested that many of the apparently 'foreign' genes may actually be 'morons', which are genes of unknown function that are carried by phages. Phages generally have a fairly limited host range, which would imply that these genes are mostly shuttling between related strains. This brings us back to Campbell's notion of accessory DNA [19]. Lan and Reeves [27] expressed much the same idea when they described the 'species genome' as a combination of 'core' and 'auxiliary' genes. We use the terms 'core' and 'accessory'.

We sequenced Rlv3841 to expose the architecture of its complex genome, and to see whether the seven replicons were specialized in their traits. In presenting our findings, we stress general trends more than individual genes, and explore the concept that the genome comprises 'core' and 'accessory' components.

## Results

### Genome organization

**Figure 1:**
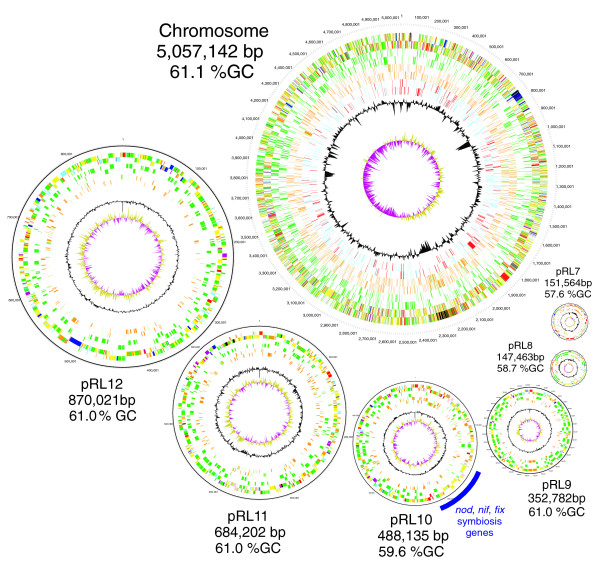
The chromosome and six plasmids of Rlv3841. The plasmids are shown at the same relative scale, and the chromosome at one-fourth of that scale. Circles from outermost to innermost indicate genes in forward and reverse orientation: all genes, membrane proteins (bright green), conserved and unconserved hypotheticals (brown conserved, pale green unconserved), phage and transposons (pink, shown for pRL7 only), and (for the chromosome only) DNA transcription/restriction/helicases (red) and transcriptional regulators (blue). Inner circles indicate deviations in G+C content (black) and G-C skew (olive/maroon). The full list of Sanger Institute standard colors for functional categories is as follows: white = pathogenicity/adaptation/chaperones (shown here in black); dark grey = energy metabolism (glycolysis, electron transport, among others); red = information transfer (transcription/translation + DNA/RNA modification); bright green = surface (inner membrane, outer membrane, secreted, surface structures [lipopolysaccharide, among others]); and dark blue = stable RNA; turquoise = degradation of large molecules; pink/purple = degradation of small molecules; yellow = central/intermediary/miscellaneous metabolism; pale green = unknown; pale blue = regulators; orange/brown = conserved hypo; dark brown = pseudogenes and partial genes (remnants); light pink = phage/insertion sequence elements; light grey = some miscellaneous information (for example, Prosite) but no function. bp, base pairs; Rlv3841, *R. leguminosarum *biovar *viciae *strain 3841.

Rlv3841 has a genome of 7,751,309 base pairs, of which 65% is in a circular chromosome and the rest are in six circular plasmids (Table 1 and Figure 1). This is consistent with earlier electrophoretic and genetic data on this strain [28]. All three rRNA operons, which are identical, and all 52 tRNA genes are chromosomal. This is in contrast to *A. tumefaciens*, *S. meliloti *and *Brucella *spp., in which some of these genes are on a second large replicon (> 1 Mb) termed a 'megaplasmid' or 'second chromosome' [3,6-8]. The chromosome of Rlv3841 (5.06 Mb) is much larger than those of *A. tumefaciens *(2.84 Mb), *S. meliloti *(3.65 Mb) and *B. melitensis *(2.12 Mb), and the total plasmid content (2.69 Mb) is also large.

### Plasmid replication genes

All six plasmids of Rlv3841 have putative replication systems based on *repABC *genes, which is the commonest system in (and apparently confined to) α-proteobacteria. RepA and RepB are thought to be a partitioning system that is essential for plasmid stability, whereas RepC is needed for plasmid replication [29,30]. Rlv3841 has the largest number of mutually compatible *repABC *plasmids yet found in one strain of any bacterial species. Although clearly homologous, each of the RepA and RepB polypeptides is highly diverged from all of the others, presumably allowing coexistence of all six plasmids (amino acid identities range from 41% to 61% for RepA, and from 30% to 43% for RepB). Most RepC sequences are also diverged (55% to 68% identity) but the pRL9 and pRL12 RepCs are 97.6% identical, suggesting that a recent recombination has taken place and that divergence of RepC is not critical for plasmid compatibility. Plasmid pRL7 has an 'extra' *repABC *operon (genes pRL70092-4) and a third version lacking *repB *(pRL70038-9).

### The distribution of different functional classes of genes

**Figure 2:**
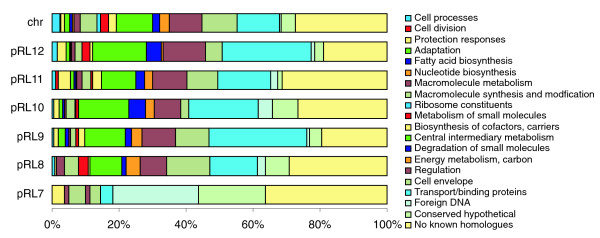
Distribution of functional classes of genes within replicons. The classes are based on those presented by Riley [86].

The chromosome and all plasmids except one (pRL7) are remarkably similar in their mix of functional classes (Figure 2). Core functions (to the left in Figure 2) are most abundant on the chromosome, but they are also strongly represented on the plasmids. The proportion of novel and uncharacterized genes ('no known homologues' or 'conserved hypothetical') is as high on the chromosome (31.5%) as it is on the plasmids (30%).

Most putatively essential genes (for example, those that encode core transcription machinery, ribosome biosynthesis, chaperones, and cell division) are chromosomal, but there are exceptions. The only copies of *minCDE*, which - although not absolutely essential for viability - are involved in septum formation and required for proper cell division [31], are on pRL11 (pRL110546-8). In *A. tumefaciens*, *S. meliloti*, and *B. melitensis*, *minCDE *are on the linear replicon, pSymB and chromosome II, respectively, raising the possibility that *minCDE *are important for segregation of large replicons other than the main chromosome. Other 'essential' genes on plasmids include major heat-shock chaperone genes *cpn10*/*cpn60 *(*groES*/*groEL*) on pRL12 (pRL120643/pRL120642), *cpn60 *on pRL9 (pRL90041), and ribosomal protein S21 on pRL10 (pRL100450). However, these genes have chromosomal paralogs, so the different copies may serve specialist functions [32] or be functionally redundant [33].

pRL7 is very different from the rest of the genome, with more than 80% of its genes being apparently foreign and/or of unknown function (Figure 2). In fact, 53 genes (28%) encode putative transposases or related proteins, and 31 (including some transposases) are pseudogenes. This plasmid appears to have accumulated multiple mobile elements, often overlapping each other. For example, gene pRL70047A (an intron maturase) is interrupted by pRL70047, which encodes a homolog of the putative transposase of the *Sinorhizobium fredii *repetitive sequence RFRS9 [34] and pRL70047D (conserved hypothetical), and the latter is in turn interrupted by an IS element (pRL70047A, B, C).

### Nucleotide composition

**Figure 3:**
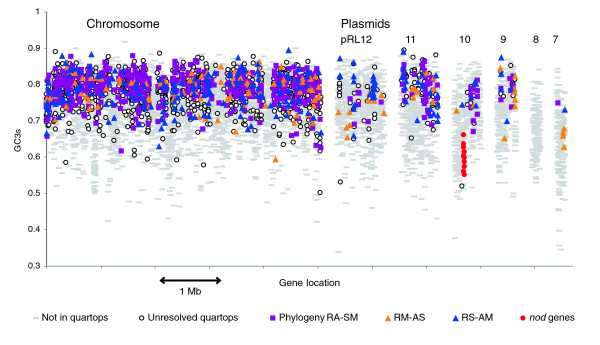
Protein-encoding genes on the chromosome and six plasmids of Rlv3841, showing their nucleotide composition. GC3s (G+C content of silent third positions of codons) is a sensitive measure of composition. Symbols indicate whether each gene encodes a quartop protein (with orthologs in *A. tumefaciens*, *S. meliloti*, and *M. loti*) and, if so, which phylogenetic topology it supports (RA-SM denotes the tree that pairs *R. leguminosarum *with *A. tumefaciens*, and *S. meliloti *with *M. loti*; RM-AS and RS-AM are similarly defined). In addition, the nodulation genes *nodOTNMLEFDABCIJ *are identified on pRL10. Rlv3841, *R. leguminosarum *biovar *viciae *strain 3841.

**Figure 4:**
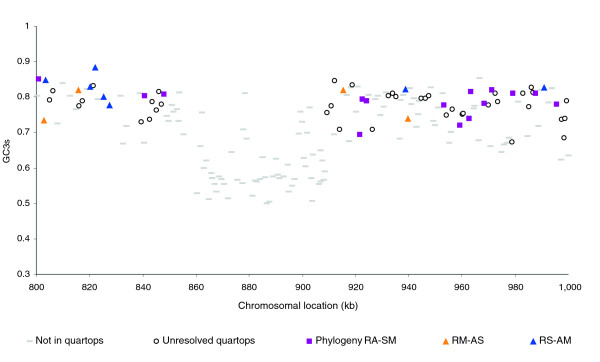
Detail of part of Figure 3, showing a chromosomal island. The island extends from 855 to 908 kilobases, genes RL0790-RL0841, and is recognizable by low GC3s (G+C content of silent third positions of codons) and absence of quartop genes. RA-SM denotes the tree that pairs *R. leguminosarum *with *A. tumefaciens*, and *S. meliloti *with *M. loti*; RM-AS and RS-AM are similarly defined.

The overall G+C content in Rlv3841 is 61% (Table 1), which is closer to that of *S. meliloti *(62%) than to that of *A. tumefaciens *(58%). Plasmids pRL10, pRL7, and pRL8 have G+C content under 60%, but the other plasmids resemble the chromosome (61%). However, these averages conceal much local variation, and a plot of GC3s (G+C content of synonymous third positions) reveals many chromosomal 'islands', in which most genes have below average GC3s (Figures 3 and 4). Several of the most distinct islands, for instance RL0790-RL0841 (54 kilobases [kb]), RL2105-RL2200 (105 kb), RL3627-3670 (52 kb) and RL3941-RL3956 (12 kb), precisely abut a tRNA gene; this suggests that they may be mobile elements that target tRNA genes, as has been described for the symbiosis island of *M. loti *[10] and many genomic and pathogenicity islands in other bacteria.

**Figure 5:**
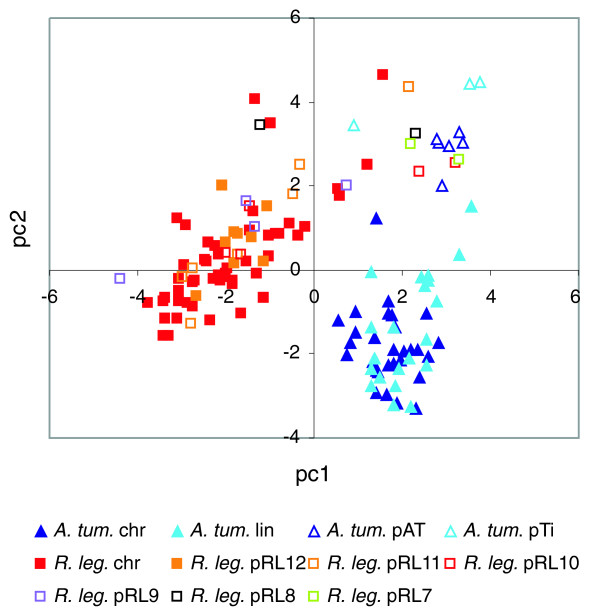
Dinucleotide compositional analysis of 100-kilobase windows of the genomes of Rlv3841 and *A. tumefaciens *C58. On the first two axes of a principal components analysis of the symmetrized dinucleotide relative abundance (DRA) of both genomes analyzed jointly, sequences from each chromosome (chr) and plasmid are identified by distinct symbols. PC1 accounts for 48.9% and PC2 for 35.6% of the total variance. Rlv3841, *R. leguminosarum *biovar *viciae *strain 3841.

Dinucleotide relative abundance (DRA) is usually thought to be relatively homogeneous within a bacterial species but different between genomes, even of close relatives [35]. The closest sequenced relative of Rlv3841 is *A. tumefaciens *C58, but the DRAs of these two genomes are consistently different, with no overlap (Figure 5). The C58 linear replicon and large parts of Rlv plasmids pRL12, pRL11, pRL10, and pRL9 have very similar compositions to their respective chromosomes, implying that they have been confined to a narrow range of hosts long enough to acquire the distinctive DRA characteristic of their host. In contrast, the DRAs of pAT, pTi, pRL7 and pRL8, as well as parts of pRL10 and pRL11, resemble each other but are very distinct from those of the corresponding chromosomes (Figure 5). Plasmids pRL7 and pRL8 are transferable by conjugation [7,17], and so they may be part of a pool of mobile replicons that have not equilibrated to the DRA of their current host genomes. Some regions of the chromosomes and larger plasmids also have this distinctive DRA, perhaps reflecting the recent insertion of 'islands' of mobile DNA. Inclusion of two more genomes, namely those of *S. meliloti *and *M. loti*, in a similar analysis does not change the overall picture (KHH and JPWY, unpublished data); the core DNA forms genome-specific clusters whereas the accessory DNA of all four genomes has similar DRA.

### A nonrandomly distributed motif

**Figure 6:**
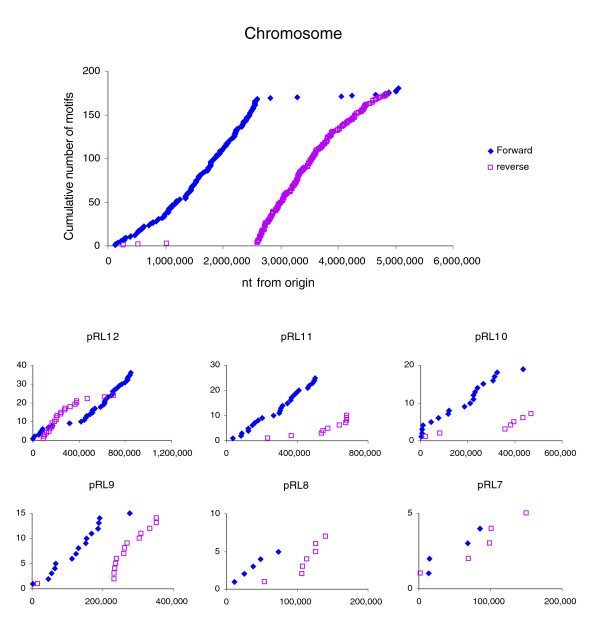
Cumulative distribution of the eight-base motif GGGCAGGG in the genome of Rlv3841. The motif is shown in forward and reverse orientation on chromosome and plasmids. Rlv3841, *R. leguminosarum *biovar *viciae *strain 3841.

The 8-base motif GGGCAGGG is much more frequent in α-proteobacterial chromosomes than expected [36]. Its orientation is biased to the leading replication strand and it is most frequent near the terminus of replication, although the reasons for this have not yet been elucidated. Its distribution on the Rlv3841 chromosome clearly illustrates this pattern (Figure 6). Of the 357 copies of the motif, 346 are oriented from origin to terminus (taking about 5,000,000 and about 2,592,000 as the presumed origin and terminus, respectively). The motif is more abundant near the terminus (approximately one every 7 kb) than near the origin (every 25 kb). A novel observation is that it also occurs on plasmids, with a similar frequency and strand bias (Figure 6). However, there is one anomaly; the motif pattern on pRL12 predicts an origin at about 400,000 rather than near *repABC*. This suggests either that replication initiation of pRL12 is not near *repABC *or that pRL12 has recently been rearranged but can survive and replicate with the 'wrong' motif distribution.

### Core genes and their phylogeny

**Figure 7:**
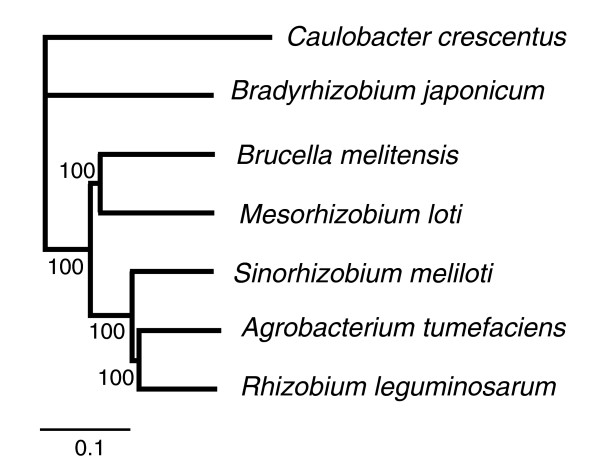
Phylogeny of completely sequenced genomes of selected α-proteobacteria. The phylogeny is based on the concatenated sequences of 648 orthologous proteins. Neighbor-Joining method with % bootstrap support indicated. Scale indicates substitutions per site.

We identified 648 Rlv3841 genes, 97% of them chromosomal, that have orthologs in each of six other fully sequenced α-proteobacterial genomes (identified in Figure 7). Overall, a phylogeny based on all of these 648 proteins (Figure 7) is consistent with the species relationships inferred from 16S ribosomal RNA, in which the closest relative of *R. leguminosarum *is *A. tumefaciens*, followed by *S. meliloti*, and then *M. loti*. However, many individual proteins actually support different phylogenetic relationships. To study this phylogenetic discordance in more detail we focused on four genomes, namely Rlv3841, *A. tumefaciens*, *S. meliloti*, and *M. loti*, which simplifies the analysis because there are just three possible topologies for an unrooted phylogeny of four organisms. We identified 2056 quartops (quartets of orthologous proteins [37]) in these four genomes (the 648 proteins above are, of course, a subset of these). The consensus topology that is implied by Figure 7 was indeed the best supported: 551 quartops supported *A. tumefaciens *as the closest relative of Rlv3841 (with > 99% posterior probability). However, 222 supported *S. meliloti *and 125 *M. loti *as the closest relative of Rlv3841 (Table 2). The remaining quartops have insufficient phylogenetic signal to support any topology with probability above 99%.

Overall, the quartops represent only 27% of the 7,263 protein-encoding genes of Rlv3841. Although 38% of chromosomal genes encode quartop proteins, only 10% of plasmid genes do so. Even among chromosomal quartops, only 66% (488/745) of those with strong phylogenetic signal support the consensus phylogeny. Replacement of the original ortholog by horizontal gene transfer may explain why so many genes, especially on plasmids, support nonconventional phylogenies. Such discordant phylogenies must have arisen from many individual events, not just a few transfers of large regions, because the genes are scattered across the genome (Figure 3).

There is a strong relationship between phylogenetic distribution and the nucleotide composition of genes. Genes in the quartops have GC3s (mean ± standard error) of 77.9 ± 0.2%, irrespective of the phylogeny that they support, but the GC3s of nonquartop genes is only 72.6 ± 0.1%.

For a broader view of gene relationships, we recorded the presence or absence of a close homolog of each Rlv3841 gene in each of the three related genomes (Table 3). There are 2,253 genes that occur in all three, 2,272 that are absent from all, and 2,740 that occur in some but not all of the other genomes (identified in Additional data file 1). The largest category in this last class comprises 546 genes that are shared by all three rhizobia but are missing from *A. tumefaciens*, which is surprising in light of the core phylogeny. Furthermore, 264 of these genes have close homologs in *Bradyrhizobium japonicum*, which shares the phenotype of root nodule symbiosis with the other rhizobia but is much more distantly related according to its core genes (Figure 7). This set of 264 genes includes, of course, the known symbiosis-related genes (discussed below under Nitrogen fixation), but we hypothesized that many of the others might have unrecognized roles in symbiosis. However, after excluding the known symbiosis genes, the representation of the Riley functional classes was not significantly different among these genes from that in the genome as a whole, and so there is no obvious evidence that they are enriched in genes that encode a particular kind of function. The Riley classes provide only a broad outline of course, especially for genomes with many genes of unknown function. However, if a significant difference existed it could readily be detected, as illustrated in the case of pRL7 (Figure 3). As more genome sequences become available there will be scope for more comprehensive analyses, which might include other measures such as the distribution of protein domain classes [38]. There is no evidence to suggest that any of these genes shared by rhizobia is directly regulated by NodD, because none of them has a *nod *box regulatory sequence. Apart from the four known *nod *boxes that regulate *nodA*, *nodF*, *nodM*, and *nodO*, the only putative *nod *boxes that we found in the genome were upstream of RL4088 and pRL120452, both of which encode putative transmembrane proteins of unknown function, but neither of which are in the rhizobium-specific gene set.

### RNA polymerase σ factors

To illustrate the differences between core and accessory genomes, we examined one group of genes that includes both core and accessory members, namely those that specify RNA polymerase σ factors. Rhizobia have many genes for RNA polymerase σ factors, and Rlv3841 is predicted to have 11 on the chromosome, one on pRL10, and two each on pRL11 and pRL12 (Table 4). In addition to the 'housekeeping' RpoD, there are two RpoH (heat shock), one RpoN (which is involved, among other things, in assimilation of certain N sources), plus other σ factors of the ECF (extracytoplasmic factor) subclass [39], only some of whose targets are known.

The chromosomal *rpoD*, *rpoN*, and *rpoH *genes exemplify the core genome, their products being highly conserved in close relatives (Table 5). Only one other σ factor gene (RL3703, *rpoZ*), which is of unknown function [40], had this pattern. In contrast, other Rlv3841 σ factor genes only occur in some of its close relatives or in none at all. One such 'Rlv-only' gene is *rpoI *(pRL120319), which encodes an ECF σ factor for promoters of the adjacent *vbs *genes, which are involved in siderophore synthesis and which are also missing from the other related genomes [41]. Thus both *rpoI *and its *vbs *'targets' are part of the Rlv accessory genome. The GC3s of the σ factor genes generally concurs with their proposed core or accessory status. Thus, *rpoD*, *rpoN*, *rpoZ*, and the two *rpoH *all have GC3s above 77%. In contrast, pRL120319 has only 64% GC3s and pRL120580 is even lower (59%). One striking exception is pRL110418, whose product (of unknown function) is absent from close relatives of Rlv but resembles σ factors in *B. japonicum *and in actinomycetes. It has higher GC3s (79%) than is typical for the Rlv accessory genome, although lower than that of the related genes in *Bradyrhizobium *(84%) or the actinomycetes (84-94%). It is possible that this is a genuinely 'foreign' gene with a composition that still reflects its origin, rather than a long-term component of the accessory genome.

### ABC transporter systems

**Figure 8:**
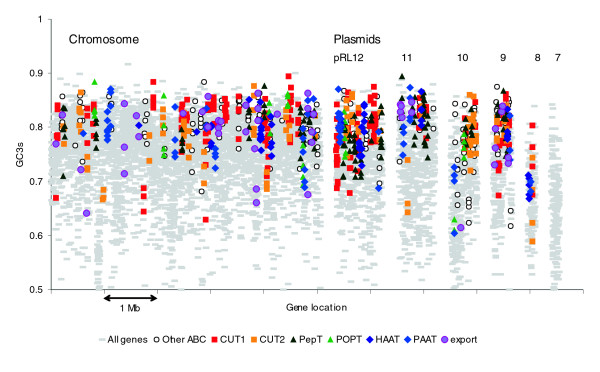
Genes on the chromosome and six plasmids of Rlv3841 encoding components of ABC transporter systems. The nucleotide composition (GC3s, i.e. G+C content of silent third positions of codons) of the components is shown. Members of the most abundant families are indicated, defined according to Saier [93]. ABC, ATP-binding cassette; Rlv3841, *R. leguminosarum *biovar *viciae *strain 3841.

Rhizobia are known to be rich in ATP-binding cassette (ABC) transporters, and there are 183 complete ABC operons in Rlv3841 (Table 5). The corresponding genes are widely distributed in the genome but they are particularly abundant on pRL12, pRL10, and especially pRL9 (Table 6 and Figure 8). In fact, they make up 27% of all genes on pRL9. Complete uptake systems contain genes for a solute binding protein, at least one integral membrane protein, and at least one ABC protein, whereas export systems do not have solute binding proteins. The total number of ABC domains is greater than the number of genes shown in Table 5 because many genes contain two fused ABC domains. For example, of the 269 ABC genes in Rlv3841, 53 are fusions yielding 322 ABC domains. There are also 19 examples of ABC domains fused to membrane protein domains. Apart from the complete operons, there are many orphan genes and gene pairs for ABC transport systems. Altogether, we have identified 816 genes that encode putative components of ABC transporters, which represent 11% of the total protein complement (see Additional data file 1 for a full list).

Only 23% of the ABC transporter genes belong to quartops (Table 6), as compared with the genome average of 38%. There are remarkable differences between the replicons in this respect; more than one-third of the transporter genes on the chromosome and pRL11 are in quartops, whereas the proportion is much lower on the other plasmids, down to a mere 7% on pRL12 (Table 6).

Given their below average representation in quartops, it is paradoxical that the transporter genes have a high average GC3s of 79.1% (genome average 74.3%). As with other genes, those in quartops have higher mean GC3s (81.1%) than those that are not (78.6%). All the genes within a particular ABC transporter operon generally have fairly similar GC3s and, with a few exceptions, the operons are in high-GC3s regions of the genome and conspicuously absent from low-GC3s islands (Figure 8).

### General metabolic pathways

*R. leguminosarum *is considered to be an obligate aerobe, and most of the genes in central metabolism are consistent with this. For example, the genome of Rlv3841 contains all of the genes for a functional TCA cycle on the chromosome (see Additional data file 3). There are actually three candidate genes for citrate synthase (RL2508, RL2509, and RL2234) on the chromosome of Rlv3841. *R. tropici *has two citrate synthase genes, one of which, namely *pcsA*, is present on its pSym and affects nodulating ability and Fe uptake [42]. The genome of Rlv3841 contains genes for isocitrate lyase (RL0761) and malate synthase (RL0054), which would allow a gloxylate cycle to operate, although strain 3841 does not grow on acetate. There are six genes whose products closely resemble succinate semialdehyde dehydrogenases (pRL100134, pRL100252, pRL120044, pRL120603, pRL120628, and RL0101), which could feed succinate semialdehyde directly into the TCA cycle. Two of these (pRL100134 and pRL100252) are on the symbiosis plasmid, and RL0101 is the characterized *gabD *gene [43]. Succinate semialdehyde is the keto acid released from 4-aminobutyric acid, an amino acid that is present at high levels in pea nodules and is a possible candidate for amino acid cycling in bacteroids. The importance of this is that amino acid cycling has been proposed to be essential for productive N_2 _fixation in pea nodules [44].

Most free-living rhizobia are believed to use the Entner-Doudoroff or pentose phosphate pathways to catabolize sugars, and to lack the Emden-Meyerhof pathway [45,46]. This is related to the absence of phosphofructokinase enzyme activity, and there appears to be no gene for this enzyme in Rlv3841. This gene has not been found in *S. meliloti *either, but it is present in *B. japonicum *(bll2850) and *M. loti *(mll5025). It has been suggested that the Emden-Meyerhof pathway does operate in *B. japonicum *[47], suggesting a fundamental difference in sugar catabolism between 'slow growing' *Bradyrhizobium *and 'fast growing' *Rhizobium *and *Sinorhizobium*. Rlv3841 has a chromosomal operon for the three genes of the Entner-Doudoroff pathway (RL0751-RL0753). In addition, there are good chromosomal candidates in *gnd *(RL2807) and *gntZ *(RL3998) for 6-phosphogluconate dehydrogenase, which is needed for the oxidative branch of the pentose phosphate pathway.

### Nitrogen fixation

The 13 *nod *genes that are known to be involved in nodulation of the host plant are tightly clustered on pRL10 (pRL100175, 0178-0189). Nearby are the *rhiABCR *genes (pRL100169-0172) that also influence nodulation [48]. These nodulation genes are surrounded by genes needed for nitrogen fixation: *nifHDKEN *(pRL100162-0158), *nifAB *(0196-0195), *fixABCX *(0200-0197), and *fixNOQPGHIS *(0205-0210A). The latter cluster has GC3s values (66-76%) that approach the genome mean (73.4%), whereas all of the other symbiosis-related genes mentioned above have strikingly low GC3s (51-66%). There is a homolog of *nodT *(pRL100291) of unknown function that is also on pRL10 but is more than 100 kb away and has much higher GC3s (72.5%).

Perhaps surprisingly, there is no *nifS *gene whose product is a cysteine desulphurase, which is believed to be involved in making the FeS clusters of nitrogenase in other diazotrophs such as *Klebsiella, Azotobacter*, and *Rhodobacter *spp. In these genera, *nifS *is closely linked to other *nif *genes, and this is also true for *nifS *in *B. japonicum *(blr 1756) and *M. loti *(Mll5865). It is not clear how the FeS clusters are made for the nitrogenases of *R. leguminosarum *and *S. meliloti *(which also lacks *nifS*). Most bacteria possess SufS, a cysteine desulphurase that is normally is involved in making the 'housekeeping' levels of FeS clusters. Interestingly, the *R. leguminosarum suf *operon has two copies *of sufS *(RL2583 and RL2578), and so these may also supply FeS for the nitrogenase protein. Alternatively, the function of NifS may be accomplished by a protein with a wholly different sequence whose identity has not yet been recognized.

*Rhizobium leguminosarum *strain VF39 has two versions of the *fixNOQP *genes, which encode the symbiotically essential *cbb*_3 _high affinity terminal oxidase [49]. Both copies are active and both copies must be mutated to give a clear effect on symbiosis [50]. Likewise, Rlv3841 has one *fixNOQP *set on pRL10 (pRL100205-0207) and another copy on pRL9 (pRL90016-0018). As in strain VF39, genes for *fixK *(pRL90019) and *fixL *(pRL90020) are upstream of *fixNOQP *on pRL9. The complexity of regulation mediated by the predicted oxygen-responsive FixK-like regulators [49] is indicated by the fact that *R. leguminosarum *has no fewer than five *fixK *homologs, three of which (pRL90019, pRL90025, and pRL90012) are on plasmid pRL9. The global regulator of *fix *genes in *R. leguminosarum *is FnrN, and this is encoded in single copy on the chromosome (RL2818), although another strain of this species, namely UPM791, has two copies [51]. The *fix *genes on pRL9 are closely linked to other genes that are involved in respiration, (for example *azuP*, pRL90021).

### Strain 3841 as a representative of the species *Rhizobium leguminosarum*

Strains within a bacterial species can differ by the presence or absence of large numbers of genes [52-54]. To date, Rlv3841 is the only sequenced strain of *R. leguminosarum*, but genetic studies of other strains have identified genes that are absent in Rlv3841, for example the pSym-borne *hup *genes for the uptake dehydrogenase system, which has been studied in some detail in another *R. leguminosarum *strain [55,56].

Rlv3841 has six plasmids, but other natural *R. leguminosarum *strains have from two to six plasmids of various sizes [11,12]. The pSym of Rlv3841 is pRL10 (488 kb), in the *repC*3 group [14], but other pSyms differ in size and replication groups [57]. Detailed genetic analysis of symbiosis in *R. leguminosarum *biovar *viciae *has focused on pRL1, a 200 kb *repC*4 plasmid [57]. The *nod *and *nif *genes of pRL1 (nucleotide accession Y00548) and pRL10 differ in just 23 nucleotides over 12 kb, which is far less than occurs between such genes of other strains of this species [58,59]. Thus, by chance, the symbiotic regions of pRL1 and pRL10 are very similar, although the plasmids are different, implying recent transfer of symbiosis genes between distantly related plasmids.

### Distinctive characteristics of each replicon

In what follows, we very briefly point out some of the salient features of each of the seven replicons.

On the chromosome, most genes have high GC3s content (Figure 3), and the DRA is fairly uniform when averaged over 100 kb windows (Figure 5). Nevertheless, the chromosome is studded with islands of genes that appear to be accessory by two criteria: their GC3s is lower and they are not in quartops (Figure 4).

pRL12, the largest plasmid, has a nucleotide composition like that of the chromosome (Figures 2 and 4) but only 9% of its genes are in quartops (Table 2), and these put Rlv3841 closer to *S. meliloti *than to the more closely related *A. tumefaciens *(Figure 7). The 23 genes supporting the *S. meliloti *relationship are in several small clusters, the largest of which (pRL120605-pRL120613; 10.7 kb) comprises an ABC transporter operon and some flanking genes that are in the same arrangement, with more than 80% amino acid identity, on pSymA of *S. meliloti*.

pRL11 is the most chromosome-like plasmid, although both the proportion of quartops and their support for the consensus phylogeny are much lower than the chromosomal values. This plasmid carries the cell division genes *minCDE*.

pRL10 has two parts which differ in composition (Figure 3). The first part of the sequence (about the first 200 genes) includes the symbiosis genes and has archetypal characteristics of the accessory genome: low GC3s and very few quartops (the known *nod *and *nif *symbiosis genes are not among the quartops, being absent from *A. tumefaciens*). The rest of pRL10 resembles the larger plasmids in being mostly high-GC3s with occasional low-GC3s islands.

pRL9 has low GC3s, with two high-GC3s regions, and few quartops. More than a quarter of the genes encode ABC transporters.

pRL8 is uniformly 'accessory', with low GC3s and no quartops.

pRL7 is an assemblage of mobile elements, dominated by repeated sequences, including many derived from phages and transposable elements. It has many pseudogenes and a short average gene length (Table 1). It has two complete, unrelated sets of replication genes and parts of a third.

## Discussion

### Why is the genome so large?

Rlv3841 has more genes than budding yeast [60] and more 'chromosomes' than fission yeast [61]. Like Rlv, many bacteria with large genomes are soil dwellers (for example, *Streptomyces*, *Bradyrhizobium*, *Mesorhizobium*, *Ralstonia*, *Burkholderia*, and *Pseudomonas *spp.). Soil is a heterogeneous environment with patchy distribution of many different substrates and potential hazards, and so a genome that encoded multiple capabilities 'just in case' would have a long-term selective advantage. The large number of transporters and regulators is consistent with this idea; a wide range of substrates can be taken up, but the pathways should be tightly regulated or the metabolic burden would be excessive. This versatility is unnecessary for rhizobia in the predictable environment of the legume nodule, but these bacteria must survive in the soil for years between these symbiotic opportunities, and their panoply of uptake systems suggests that they are often metabolically active during this phase. However, omnivory is not the only possible strategy for survival in soil; *Nitrosomonas*, for example, is a specialist in NH_3 _oxidation and has a genome of just 2.8 Mb [62].

### Is there a core genome and an accessory genome?

Our concept of the accessory genome is of a pool of genes that have a long-term association with a bacterial species or group of species but are adapted to a nomadic life. An accessory gene moves readily between strains but will be found in only a subset of strains at any one time. Each bacterial genome we look at has many genes that are not seen elsewhere ('ORFans' [63]) or that have a sporadic distribution in other known genomes. These are often interpreted as newly acquired genes, particularly if their composition is not typical of the genome, but we suggest that many may in fact have a long-term association with the species but a patchy distribution across strains. There is a parallel between this concept and the view of phages as the source of a pool of potentially adaptive genes [26], because each phage generally has a limited host range. In this section we interpret the Rlv3841 genome in the light of these ideas.

Our survey of the genes of Rlv3841 considered their location, function, composition, and phylogeny. The genome is heterogeneous in all four respects, but patterns emerge when they are considered jointly. The archetypal 'core' genes are on the chromosome, have an essential function in cell maintenance, have a high G+C content, and have orthologs in related species that conform in their relationships with the species phylogeny deduced from rRNA sequences. All of these properties are positively correlated, and some genes exhibit all of them. However, these are surprisingly few (1,541), accounting for less than one-third of the genes on the chromosome.

At the other end of the spectrum are archetypal 'accessory' genes that are located on plasmids, that confer sporadically useful adaptations, that are lower in G+C, and that are missing from some related genomes or, if present, exhibit phylogenetic evidence for horizontal transfer. The *nod *genes illustrate all of these points, but there are hundreds of other genes with similar properties, their annotated functions varying from fairly specific (sugar transporter, response regulator) to 'conserved hypothetical' or 'no significant database hits'. We know the exact functions of most *nod *genes [1], but this reflects many years of study without which the *nod *region would have been just one more tract of vaguely annotated genes. Indeed, some of the genes would probably have attracted misleading annotations, such as 'involved in chitin synthesis' for *nodC *[64]. We presume that many other accessory genes are like the *nod *genes in having specific functions that relate to lifestyle, and that we may eventually elucidate these.

The low GC3s of the accessory genes (Figure 3), also reflected in their distinctive DRA (Figure 5), marks them as different from the core genome. Such genes occur in most sequenced genomes, and are usually regarded as recent arrivals via horizontal gene transfer from a donor whose composition they are presumed to reflect [20]. However, a consideration of the Rlv3841 genome challenges this view. First, acquisition from arbitrary donors cannot explain the relatively consistent composition of the accessory genome (Figure 5); many segments of *R. leguminosarum *plasmids resemble the *Agrobacterium *plasmids in composition, but none resemble the *Agrobacterium *chromosomes. Even more compellingly, *nod *genes consistently have lower GC3s than is typical for the host genome in any of the wide range of both α-proteobacteria and β-proteobacteria that are known to carry them [65,66], and those of Rlv3841 conform to this pattern (Figure 3).

The hypothesis that the composition of accessory genes reflects the genomic norm of their donor would require that there is an unknown 'real rhizobium' out there, with low genomic GC3s, that has relatively recently donated the *nod *genes to all the diverse nodulating bacteria currently known. This seems highly implausible, and in fact a recent radiation is ruled out by the high sequence divergence among homologous *nod *genes [67]. Instead, the example of the nodulation genes points to the existence of an accessory gene pool that maintains a distinctive composition despite an apparent long-term cohabitation with core genomes that equilibrate to quite different compositions. The age of the common ancestor of nodulated legumes has been estimated at 59 million years [68], and the nodulation (*nod*) genes that determine host specificity have probably been diverging for a similar length of time. Although this has been long enough for sequence divergence in excess of 60% [69], *nod *genes are certainly much more recent than the common ancestor of all of the bacteria that currently carry them; *Rhizobium *and *Bradyrhizobium *are thought to have been separated for 500 million years [70] and the β-proteobacterial symbionts are, of course, more distant still. The *nod *genes are therefore indisputably part of the horizontally transferred accessory gene pool.

The distinction between core and accessory genomes is well illustrated by the large Rlv3841 genome with its multiple replicons, but this appears to be a widespread property of bacterial genomes [26]. Genes in *E. coli *that have no known homologs in other genomes (for instance, ORFans) are short, have low G+C and evolve rapidly, but they ameliorate to the core genome composition very slowly [71]. We can only speculate on whether the distinctive composition of accessory genes is due to selection for properties favorable for gene transfer, or to different mutational bias, perhaps reflecting a history of replication in plasmids or phages rather than chromosomes. Low G+C is typical of genes in phages, including 'morons' [26], which are not needed for phage functions and may be thought of as host accessory genes in transit. A low G+C content may make DNA less susceptible to attack by restriction enzymes [26] and more likely to undergo recombination [72]. Phages appear to be the major vectors of the accessory gene pool in *E. coli *and are probably significant in rhizobia too, but large transmissible plasmids such as pRL7 and pRL8 are common in rhizobia and probably play an important role. Not all accessory genes are currently on plasmids but their location varies between genomes, and so it is plausible that any accessory gene will have spent part of its recent history on plasmids, as supposed by Campbell [19]. The chromosome of Rlv3841 has many distinct 'islands' of genes with typical accessory characteristics: low GC3s and a sporadic phylogenetic distribution (Figure 3). This subset is rich in genes of unknown function. These are part of the accessory genome residing, probably temporarily, in the chromosome.

More enigmatic are the many genes that are not in quartops, which indeed are often unique to Rlv3841, and yet have the typical composition of the core genome. These are not readily classified as either typical accessory or core genes but may form a third category. Possible examples are the σ factor genes pRL100385, pRL110418, and pRL110007, which all have high GC3s but no orthologs in *S. meliloti *or *A. tumefaciens*, and many of the ABC transporter genes (Figure 8). Genes with these characteristics are particularly abundant on the large plasmids of Rlv3841 (Figure 3), and unrelated genes with similar characteristics are found on the second chromosomes of *Agrobacterium *and *Brucella *and pSymB of *Sinorhizobium *[3,6-8]. Their composition implies a long-term relationship with their host genome, but their sporadic distribution suggests that they confer special, perhaps strain-specific or species-specific, adaptations rather than core functions.

Although there are consistent average differences between the core and (one or more) accessory classes of genes, the dividing line is not clear cut. There is a continuous, unimodal distribution of GC3s values (Figure 3), and the designation of 'core' depends on the choice of genomes for comparison. Furthermore, there are some accessory genes, such as pRL110418 (for the actinomycete-like σ factor) that appear to be genuinely 'foreign' and do not have the composition of the long-term accessory genome. The notion of distinct categories is, of course, an oversimplification, but it provides a framework for understanding the organization of the genome.

### Questions that the genome raises about bacterial function, evolution, and ecology

The genome of Rlv3841 is a snapshot of one example of an *R. leguminosarum *genome. We know that the number and size of plasmids, and the complement of genes, differ in other isolates of the species. Now that we have so many snapshots of bacterial genomes, the challenge is to elucidate the assembly rules. What is constant and what is ephemeral? Which parts of the accessory genome are species specific, and which range more widely? If accessory genes are adapted to a nomadic life in which they perpetually move between genomes, how is their regulation integrated into the host cell? The fact that Rlv3841 shares more genes with the other rhizobia *S. meliloti *and *M. loti *than with its closer relative *A. tumefaciens *(Table 3) supports the idea that the accessory genome is related to lifestyle and that accessory genes can confer different ecologic niches on similar core genomes. This conclusion must be tempered, though, by the realization that bacteria can be multifunctional and isolates that successfully combine rhizobial and agrobacterial properties have been found [73].

If accessory genes are sporadically distributed and mobile, then similarity in the core genome does not guarantee that bacteria are similar in ecology (for example, *R. leguminosarum *and *A. tumefaciens*). In the face of a transient accessory genome, does a genomic species have sufficient ecologic coherence to maintain its identity? This is an important question in relation to our understanding of the nature of bacterial species. Cohan has argued that periodic selection purges variation among ecologically equivalent bacteria, and this creates the genetic coherence that allows species to be defined [74]. However, if an ecologic niche is conferred by the accessory genome then it will not be tightly correlated with the core genome, which includes the ribosomal RNA and other genes typically considered to define species. For bacteria with large accessory genomes, genetic processes (homologous recombination) may be more important than ecologic processes (periodic selection) in maintaining coherence of the core genome of the species.

A related issue concerns the numerous surveys of bacterial diversity based on libraries of ribosomal RNA genes [75]. Although these have been very successful in expanding our awareness of microbial communities, the ecologic significance of the diversity [76] is more difficult to establish because of the weak linkage between core genome and accessory adaptations. On the other hand, metagenomic studies give us access to the ecologically important accessory genes (if only we knew how to read them!) but, except in the simplest communities, we do not know with which core genomes they are associated [77].

## Conclusion

The genome of *R. leguminosarum *strain 3841 is unusual in having six large plasmids in addition to a large chromosome. At least two-thirds and perhaps more than 90% of its 7,263 genes must be considered accessory because they are not universally shared even among closely related species. The core, putatively essential, genes have a characteristic high G+C composition, whereas the accessory genes range from those that share this core-like composition, such as many ABC transporter genes, to those that have much lower G+C, including most known symbiosis-related genes. Accessory genes are more frequent on the plasmids, but are also found in genomic islands scattered through the chromosome. They surely hold the key to unraveling bacterial adaptation and specialization, but for the moment the great majority remain of unknown function.

Bacteria with large complex genomes are abundant and important in nature, and so this genome of Rlv3841 should serve as a further reference point for developing our understanding in many directions.

## Materials and methods

### Sequencing strategy and annotation

Rlv3841 [15,16] was grown to mid-log phase in TY medium [78] and genomic DNA was isolated using a Blood and Cell Culture DNA midi kit (Qiagen, Hilden, Germany). DNA was sonicated and size selected on an agarose gel, and used to make libraries in pUC18, pMAQ1b, m13MP18, and pBACe3.6.

The final assembly was based on 60,600 paired end-reads from a pUC18 library (insert sizes 3.0-3.3 kb) and 54,700 paired end-reads from a pMAQ1b library (inserts 5.5-6.0 kb), with a further 9,000 single reads from an m13mp18 library to give 7.6-fold sequence coverage of the genome. To produce a scaffold, 2520 reads were generated from a 23-48 kb insert library in pBACe3.6, giving about 5.8× clone coverage. Sequencing was done using Amersham Big-Dye (Amersham, UK) terminator chemistry on ABI3700 sequencing machines. The sequence was assembled with Phrap (Green P, unpublished data) and finished using GAP4 [79]. To finish the sequence, approximately 13,400 extra reads were generated to fill gaps and ensure that all bases were covered by reads on both strands or with different chemistries. All identified repeats were bridged using read-pairs or end-sequenced polymerase chain reaction products. The three rRNA operons were identical; whole-genome shotgun reads did not indicate any polymorphisms, and each operon was independently sequenced from BAC clones or long-range polymerase chain reaction products to confirm this.

The finished sequence was annotated using Artemis software [80]. Initial coding sequence predictions were performed using Orpheus and Glimmer2 software [81,82]. The two predictions were amalgamated, and codon usage, positional base preference, and comparisons with nonredundant protein databases using BLAST and FASTA software were used to curate the predictions. The entire DNA sequence was also compared in all six reading frames against nonredundant protein databases, using BLASTX, to identify all possible coding sequences. Protein motifs were identified with Pfam and Prosite, transmembrane domains with TMHMM [83], signal sequences with SignalP [84], rRNA genes with BLASTN, and tRNAs with tRNAscan-SE [85]. Functional assignments, EC numbers, and other information, including functional classes in the Riley classification [86], were all manually curated. Artemis Comparison Tool [87] was used to visualize BLASTN and TBLASTX comparisons between genomes. Pseudogenes had one or more inactivating mutations, which were checked against the original sequencing data.

The data have been submitted to the EMBL database (accession numbers AM236080 to AM236086).

### Bioinformatic analyses

Quartets of orthologous proteins (quartops) were sets of reciprocal top-scoring BLASTP hits (putative orthologs) in all pairwise genome comparisons between *R. leguminosarum*, *S. meliloti*, *M. loti*, and *A tumefaciens *[37]. A narrower 'core' was similarly defined using additional comparisons with *B. japonicum*, *Brucella melitensis*, and *Caulobacter crescentus*. Genome sequences for these comparisons were downloaded from GenBank. For each quartop, the three posterior probabilities were calculated with MrBayes version 3 [88].

Symmetrized DRA frequencies were calculated in each replicon for nonoverlapping windows of varying sizes using custom-made Perl scripts. This calculation is based on an odds ratio, which considers the observed frequency divided by the expected frequency of each dinucleotide [22,35,89-91]. Symmetrized frequencies were obtained by concatenating the original sequence with its inverted complementary sequence; this reduced the 16 original dinucleotides to 10 and controlled for strand bias, allowing for the comparison of different species. This analysis results in a 10 × *n *matrix, where *n *is the number of genomic windows. Principal components analysis was carried out in MATLAB version 6.5 release 13 using the MVARTOOLS toolbox. The percentage variance represented by each principal component was assessed through the construction of a scree plot. With 84.5% of the total variance explained by principal components 1 and 2, these axes alone were deemed sufficient for analysis (PC1 48.9% and PC2 35.6%).

GC3s content of individual genes was calculated using CODONW [92].

## Additional data files

The following additional data are included with the online version of this article: A text file containing a tab-separated table of all of the protein-encoding genes with Riley functional class, location, GC3s, peptide length, quartop membership, presence/absence of homologs in related species, and (where appropriate) ABC transporter classification (Additional data file 1); a text file containing a key to Riley functional classes (Additional data file 2); and a text file containing a list of genes that encode central metabolic pathways (Additional data file 3).

## Supplementary Material

Additional data file 1 A text file containing a tab-separated table of all of the protein-encoding genes with Riley functional class, location, GC3s, peptide length, quartop membership, presence/absence of homologs in related species, and (where appropriate) ABC transporter classification. Click here for file

Additional data file 2 A text file containing a key to Riley functional classes. Click here for file

Additional data file 3 A text file containing a list of genes that encode central metabolic pathways. Click here for file

## Figures and Tables

**Table 1:** Genome statistics for Rlv3841

Replicon	Base pairs	Percentage G+C	Protein-encoding genes	Percentage Coding	Mean protein length (aa)	rRNA operons	tRNA genes
Chromosome	5,057,142	61.1	4,736	86.3	309	3	52
pRL12	870,021	61.0	790	90.3	335		
pRL11	684,202	61.0	635	87.5	318		
pRL10	488,135	59.6	461	81.7	304		
pRL9	352,782	61.0	313	88.8	337		
pRL8	147,463	58.7	140	83.4	306		
pRL7	151,546	57.6	188	74.6	224		
Total	7,751,309	60.86	7,263	86.4	309	3	52

aa, amino acids; Rlv3841, *Rhizobium leguminosarum *biovar *viciae *3841.

**Table 2:** Phylogenies supported by quartets of orthologous proteins shared between Rlv3841, *A. tumefaciens*, *S. meliloti*, and *M. loti*

	Total proteins	Number in quartops	Percentage in quartops	Phylogeny supported		
				
				RA-SM^a^	RS-AM	RM-AS
Chromosome	4,736	1,798	38.0	488	165	92
pRL12	790	70	8.9	7	23	10
pRL11	635	124	19.5	36	22	10
pRL10	461	28	6.1	10	6	2
pRL9	313	30	9.6	9	5	7
pRL8	140	0	0	0	0	0
pRL7	188	6	3.2	1	1	4
All	7,263	2,056	28.3	551	222	125

^a^Number of quartops supporting the phylogeny ([*R. leguminosarum*, *A. tumefaciens*], [*S. meliloti*, *M. loti*]) with at least 99% probability (and likewise for the other two possible topologies). Rlv3841, *Rhizobium leguminosarum *biovar *viciae *3841.

**Table 3:** Numbers of genes unique to Rlv3841 or shared with one or more related genomes

Distribution^a^	Chromosome	pRL12	pRL11	pRL10	pRL9	pRL8	pRL7	All replicons
R	1,145	293	276	214	128	91	123	2,270
R+A	236	88	34	46	28	15	21	468
R+S	267	61	42	36	25	5	9	445
R+M	280	78	50	41	27	3	11	490
R+A+S	347	67	37	20	19	3	6	499
R+A+M	170	47	19	17	19	17	3	292
R+S+M	365	68	33	43	25	6	6	546
R+A+S+M	1,926	88	144	44	42	0	9	2,253
Total genes	4,736	790	635	461	313	140	188	7,263
(R+S+M)+B^b^	183	25	12	26	10	5	4	264

^a^Genes of *Rhizobium leguminosarum *biovar *viciae *3841 (Rlv3841) are classified by whether they are unique to this genome (R) or have homologs in the genomes of *A. tumefaciens *(A), *S. meliloti *(S), or *M. loti *(M). ^b^Of the 546 genes shared by the three rhizobia (R+S+M) but missing from A, 264 had homologs in *B. japonicum*, a distantly related rhizobium. Rlv3841, *Rhizobium leguminosarum *biovar *viciae *3841.

**Table 4:** The σ factors of Rlv3841

Gene and name (if known)^a^	GC3s, quartop^b^	Closest homologs^c^		
		
		Species	Gene	BLAST e value
RL0422 (RpoN, used for N assimilation)	0.791 Y	*S. meliloti*	SMc01139	0.0
		*A. tumefaciens*	AGR_C_581	0.0
		*M. loti*	mll3196	2E-161
		*Brucella suis*	BR0158	3E-156
RL0788	0.732 N	*Pseudomonas fluorescens*	Pflu02001224	9E-56
		*Erwinia carotovora*	ECA2017	E-43
		*Burkholderia ambifaria*	BambDRAFT_3017	9E-30
		*S. meliloti*	SMa01432	2E-27
		*A.tumefaciens*	AGR_C_2739	2E-16
		*M.loti*	mlr77732	E-16
RL1138	0.714 N	*Ralstonia eutropha*	Reut_B3794	E-47
		*Bradyrhizobium japonicum*	bll6484	2E-44
		*Silicibacter pomeroyi*	SPO31254	E-29
		*M. loti*	mlr7773	7E-29
		*A. tumefaciens*	AGR_C_2739	2E-28
		*S. meliloti*	SMc01419	9E-23
RL3020	0.749 Y	*A. tumefaciens*	AGR_C_4121	6E-56
		*B. suis*	BRA0021	2E-32
		*Burkholderia fungorum*	Bcep02004893	7E-32
		*Caulobacter crescentus*	CC3253	2E-30
		*M. loti*	mll3493	4E-25
		*S. meliloti*	SMb20531	2E-18
RL3235	0.777 N	*S. meliloti*	SMc01419	E-48
		*A. tumefaciens*	AGR_C_3679p	9E-44
		*Sphingopyxis alaskensis*	SalaDRAFT_1954	5E-23
		*B. japonicum*	bll2628	6E-23
		*M. loti*	mlr04075	E-18
RL3402 (RpoD, 'housekeeping' σ)	0.823 Y	*A. tumefaciens*	AGR_C_3929p	0.0
		*S. meliloti*	SMc01563	0.0
		*B. suis*	BR1479	0.0
		*M. loti*	mll2466	0.0
RL3509	0.758 N	*S. meliloti*	SMc02713	2E-38
		*S. alaskensis*	SalaDRAFT_1954	4E-18
		*Burkholderia cepacia*	Bcepa03005434	5E-18
		*Novosphingobium aromaticivorans*	DSM 12444	2E-17
		*A. tumefaciens*	AGR_C_3679	E-14
		*M. loti*	mlr7773	5E-12
RL3703 (RpoZ)	0.790 Y	*S. meliloti*	SMc01506	5E-84
		*B. suis*	BR1658	3E-80
		*Bartonella henselae*	BH13830	5E-73
		*A. tumefaciens*	AGR_L_1386p	5E-68
		*M. loti*	mll3697	7E-68
RL3766 (RpoH, heat shock σ)	0.794 Y	*A. tumefaciens*	AGR_C_4439p	2E-155
		*S. meliloti*	SMc00646	3E-148
		*M. loti*	mlr3741	5E-138
		*B. suis*	BR1650	E-133
RL4524	0.780 N	*M. loti*	mlr8088	5E-38
		*Rhodospirillum rubrum*	Rrub02003750	3E-37
		*Rhodobacter sphaeroides*	'rpoE'	3E-34
		*Magnetospirillum magnetotacticum*	Magn03011107	E-33
		*S. meliloti*	None listed in top 50 matches	
		*A. tumefaciens*	None listed in top 50 matches	
RL4614 (RpoH2, heat shock σ)	0.771 N	*S. meliloti*	SMc03873	5E-118
		*M. loti*	mlr3862	2E-98
		*B. suis*	BR1763	5E-97
		*B. henselae*	BH15210	3E-95
		*A. tumefaciens*	AGR_C_4439p^d^	7E-58
pRL10385	0.720 N	*M. loti*	mll1224	2E-52
		*Microbulbifer degradans*	Mdeg02003584	E-28
		*R. sphaeroides*	Rsph03003515	E-27
		*Solibacter usitatus*	AcidDRAFT_4778	6E-22
		*S. meliloti*	None listed in top 50 matches	
		*A. tumefaciens*	None listed in top 50 matches	
pRL11007 (EcfR)	0.778 N	*M. loti*	mlr0407	4E-57
		*R. sphaeroides*	Rsph03002607	E-45
		*M. magnetotacticum*	Magn03006880	8E-39
		*Bordetella parapertussis*	BPP1584	4E-37
		*A. tumefaciens*	AGR_L_1386p	3E-24
		*S. meliloti*	SMa0143	2E-22
pRL110418	0.794 N	*B. japonicum*	blr7812	2E-83
		*Streptomyces avermitilis*	SAV7424	7E-74
		*Nocardia farcina*	nfa4970	3E-73
		*Frankia *sp.	Francci3DRAFT_3517	7E-66
		*M. loti*	mll6869	8E-33
		*S. meliloti*	None listed in top 50 matches	
		*A. tumefaciens*	None listed in top 50 matches	
pRL120319 (RpoI, iron uptake sigma)	0.640 N	*Azotobacter vinelandii*	AvinDRAFT_4065	4E-20
		*Pseudomonas aeruginosa*	'pvdS'^e^	3E-18
		*S. meliloti*	SMc04203	8E-09
		*A. tumefaciens*	AGR_pAT_442	2E-08
pRL120580	0.587 N	*Rhodopseudomonas palustris*	RPA0550	4E-40
		*R. rubrum*	Rrub02003750	8E-38
		*M. magnetotacticum*	Magn03011107	5E-37
		*Pseudomonas syringae*	PSPTO1043	3E-28
		*M. loti*	mlr8088	4E-26
		*A. tumefaciens*	AGR_C_3605	6E-14
		*S. meliloti*	None listed in top 50 matches	

^a^*Rhizobium leguminosarum *biovar *viciae *3841 (Rlv3841) locus tags are shown and, if the gene has been named in *R. leguminosarum*, this name is shown. ^b^GC3s (G+C content of silent third positions of codons), member of quartop (yes [Y]/no [N]). ^c^Each of the Rlv3841 sigma factors was used in BLASTP searches (maximum of 50 matches). The sequences and the locus tag numbers, together with the 'e' values, are shown for the top four matches. In those genera (*Brucella*, *Pseudomonas*, *Streptomyces*, *Burkholderia*) with more than one sequenced species, only one example is shown for each genus. In those cases in which one or more of the species used in the quartop analysis is not in the top four matches, these are also included. ^d^*A. tumefaciens *has only one *rpoH *gene (AGR_C_4439p) whereas *R. leguminosarum*, *M. loti*, and *S. meliloti *have two. AGR_C_4439p is more closely related to Rlv3841 RpoH1 (RL3766) than to Rlv3841 RpoH2 (RL4614). ^e^Also known as PfrI in *P. putida*, and PbrA in *P. fluorescens*.

**Table 5:** Classification of ABC transporters of Rlv3841 compared to those of *S. meliloti *1021

Family	*R. leguminosarum*		*S. meliloti*	
	
	ABC genes	Complete systems	ABC genes	Complete systems
Uptake systems				
CUT1	46	36	29	26
CUT2	35	29	37	37
FeCT	5	3	5	5
FeT	2	2	1	1
HAAT	22	11	10	5
MolT	1	1	1	1
MZT	3	3	2	2
NitT	11	5	4	4
PAAT	16	13	16	13
PepT	49	30	33	25
PhnT	3	1	1	1
PhoT	2	2	1	1
POPT	8	7	12	12
QAT	6	5	5	4
SulT	1	1	2	1
TauT	0	0	1	1
ThiT	2	0	1	1
Unclassified	26	13	18	6
Efflux systems				
Export	31	21	21	18
Total	269	183	200	164

Families are defined according to Saier [93]. Complete uptake systems possess at least one solute binding protein, one ATP-binding cassette (ABC), and one integral membrane protein. Complete export systems need only possess one ABC and one integral membrane protein.

**Table 6:** Distribution and phylogeny of ABC transporter genes

	Transporter genes	Percentage of all genes	Number in quartops	Percentage in quartops	Phylogeny supported		
					
					RA-SM^a^	RS-AM	RM-AS
Chromosome	383	8.1	128	33.4	31	20	16
pRL12	172	21.7	12	7.0	0	9	0
pRL11	77	12.1	29	37.7	11	3	1
pRL10	83	18.0	8	9.6	5	1	0
pRL9	86	27.5	14	16.3	3	3	4
pRL8	15	10.6	0	0	0	0	0
pRL7	0	0	0	0	0	0	0
All	816	11.2	191	28.3	50	36	21

^a^Number of quartops supporting the 'core' phylogeny ([*R. leguminosarum*, *A. tumefaciens*], [*S. meliloti*, *M. loti*]) with at least 99% probability (and likewise for the other two possible topologies). ABC, ATP-binding cassette.
